# Clinician perspectives on having point of care tests made available to them during out of hours home visiting

**DOI:** 10.1186/s12875-021-01571-0

**Published:** 2021-12-16

**Authors:** S. Dixon, M. Glogowska, S. Garland, H. Hunt, D. Lasserson, G. Hayward

**Affiliations:** 1 Nuffield Department of Primary Care Health Sciences, Radcliffe Observatory Quarter, Woodstock Road, Oxford, OX2 6GG England UK; 2 Oxford Health Foundation Trust, Warneford Hospital, Warneford Lane, Headington, Oxford, OX3 7JX England UK; 3 Warwick Medical School, The University of Warwick, Coventry, CV4 7AL England UK

**Keywords:** Point of care tests (POC) tests, Near patient tests, Primary care urgent care, Out of hours (OOH), Home visit

## Abstract

**Background:**

Little is known about clinicians’ perspectives on the use of point of care (POC) tests in assessment of acute illness during primary care out of hours (OOH) care. During a service improvement project, POC tests (including creatinine, electrolytes, haemoglobin and lactate) were made available to clinicians undertaking OOH home visits, with the clinicians allowed absolute discretion about when and whether they used them.

**Method:**

To explore clinicians’ perspectives on having POC tests available during OOH home visits, we undertook a qualitative study with clinicians working in Oxfordshire OOH home visiting teams.

We conducted 19 Semi-structured interviews with clinicians working in OOH, including those who had and had not used the POC tests available to them. To explore evolving perspectives over time, including experience and exposure to POC tests, we offered clinicians the opportunity to be interviewed twice throughout the study period. Our sample included 7 GPs (4 interviewed once, 3 interviewed twice - earlier and later during the study), 6 emergency practitioners (EPs) including advanced nurse practitioners and paramedics, 1 Healthcare Assistant, and 2 ambulatory care physicians. Interviews were audio-recorded, transcribed verbatim and analysed thematically.

**Results:**

The clinicians reflected on their decision-making to use (or not use) POC tests, including considering which clinical scenarios were “appropriate” and balancing the resources and time taken to do POC tests against what were perceived as likely benefits. The challenges of using the equipment in patients’ homes was a potential barrier, though could become easier with familiarity and experience. Clinicians who had used POC tests described benefits, including planning onward care trajectories, and facilitating communication, both between professionals and with patients and their families.

**Conclusion:**

Clinicians described a discriminatory approach to using POC tests, considering carefully in which situations they were likely to add value to clinical decision-making.

## Background

Current healthcare policy for the NHS in England seeks to treat more people in their homes, as an alternative to hospitalisation [1], thus shifting the balance of care to the community. Implementing initiatives which transfer care from hospital to other settings requires consideration of organisational and individual factors which determine their feasibility and effectiveness [2]. Point of care (POC) testing is identified as a technology that could facilitate community-based care [3].

An intervention which could enable the expansion of acute care capabilities in the community is improved access to diagnostic technologies previously only available in hospital settings. POC blood testing technology is increasing in scope and reducing in size, meaning that it is now possible for clinicians to have immediate access to some blood test results in patient’s homes. This technology is a relatively expensive additional tool which has not previously been available to clinicians undertaking urgent care out of hours home visits.

Out of Hours (OOH) Primary Care involves high risk decision making, as clinicians assess patients who are more likely to have acute illness [4–6], without prior knowledge of the patient and with limited access to diagnostic tests or medical records. For older patients living with frailty who develop acute illness, assessments by OOH clinicians are frequently made in patients’ homes [7], making this an ideal setting to trial point of care blood tests. Research suggests that clinicians working in OOH want access to more POC diagnostic tests [8], but how and when they choose to use them and how they incorporate it into their consultations has not been previously described.

In a recent mixed methods service improvement project, point of care (POC) tests were made available to clinicians working in a Primary Care OOH home visiting service in Oxfordshire, UK. In this setting, the out of hours service is contractually provided by a community health trust. Clinician’s work shifts to provide and cover the out of hours service. This includes a range of clinicians (general practitioners (GPs), advanced nurse practitioners (ANPs) and paramedics or extended care practitioners (ECPs), who might work in out of hours as their only clinical role or as an adjunct to an in-hours or other clinical role. Clinicians within this out of hours service may work regular shifts or adhoc sessions, with a range in how often they work (for example more than weekly to less than monthly). Senior clinicians from the out of hours service were involved in designing and approving the service innovation. All clinicians working in the out of hours service had complete discretion and choice about when or whether they utilised the POC tests or equipment, which was made available without protocols or guidance beyond how to use the equipment. Uptake of POC test use was low, used in only 2% of home visits, with almost two thirds of tests done by Emergency Practitioners (EPs) [9]. The qualitative component of this project offered an opportunity to understand clinicians’ perspectives and experiences of using POC tests where they were made available without their use being proscribed by clinical or trial protocols.

## Method

Within the project, the qualitative component was of particular significance, as this is an area where little is known and a directly pertinent literature is lacking. Qualitative research is highly appropriate for capturing and exploring people’s experiences and perceptions of phenomena [10] – in this case the use of POC tests by OOH clinicians.

### Details of the POC test service improvement project

Clinicians undertaking OOH primary care home visits had access to an Abbott i-stat machine, which is a compact device weighing approximately 500 g and measuring 20 × 6 × 5 cm. Two panels of tests were available; CHEM 8 (sodium, potassium, calcium, glucose, creatinine, total carbon dioxide, anion gap, urea and haemoglobin), and CG4 (lactate and blood gases). Training on using the equipment was provided by the manufacturer and cascaded to staff. Clinicians undertaking home visits were able to choose whether and when the POCT were used. There was no specific clinical training, protocols, or guidance about test usage. Clinicians were able to access advice through usual service routes, which included speaking with other health-care professionals working in the service or calling secondary care teams for advice. A full description of the usage of tests in the service improvement project has been published previously [9].

### Recruitment

The Research and Development Department of Oxford Health NHS Foundation Trust reviewed the protocol and prospectively approved the clinician interviews as part of the service evaluation linked to this service improvement project. Informed consent was obtained from all participants.

Clinicians who carried out OOH home visits in the participating OOH sites were invited to take part in a qualitative interview by a member of the clinical team. Clinicians were free to choose whether or not to participate, received an information sheet and gave their written consent to be interviewed, including consent for publication of the project findings and the use of written quotations. Clinicians who chose to participate were offered £30 in gift vouchers.

We interviewed GPs, paramedics, advanced nurse practitioners, ambulatory care unit clinicians and a Health Care Assistant about their experiences of having POCT made available to them to explore:

- *Their views on having POCT available to them during home visits to patients*
- *Their decision-making about whether (or not) to use the test equipment*
- *What they perceived as facilitators and barriers to using POCT during home visits*

### Data collection

During November 2016 – December 2017 semi-structured, individual interviews were conducted with practising OOH clinicians. This method of data collection is highly appropriate for capturing people’s experiences and perceptions and has considerable power to explain actions, decisions and processes.

Interviews were completed face-to-face or via the telephone by SD (GP and researcher), according to the preference of the clinician. SD is a GP who works in the locality served by the out of hours service but does not work for this team and has no personal or professional experience in POC blood tests. The participants were made aware that she was a GP. None of the participants were known personally or professionally to the researchers. The analysis was co-conducted with an experienced health services researcher and analysis and interpretation were reviewed and developed with input from the study team (MG). We spoke to three clinicians twice throughout the implementation period in the early and later stages of the study period. An initial topic guide was developed from the literature and the experiences of clinicians in our research team, which was used flexibly as the interviews proceeded. The interviews were audio-recorded, transcribed verbatim and analysed thematically.

### Data analysis

Analysis was led by SD (GP and researcher) and guided by the constant comparative method, which included reading and familiarisation with the transcripts, noting and recording initial themes and then conducting systematic and detailed open coding using QSR NVivo 11, a qualitative data analysis software package which assists in the organisation and retrieval of data.

The coding of the first set of interviews generated an initial coding framework, which was discussed with the members of the research team. The study design was iterative so that the initial analysis guided ongoing sampling, data collection and further analysis. The initial coding framework was further developed and refined as analysis proceeded. Throughout the analysis process, the research team critically discussed ideas for categories and themes which emerged from the data. This ensured that our findings were credible, dependable and firmly grounded in the data [10].

## Results

Nineteen interviews were undertaken with clinicians working in the participating OOH home visiting service (Table 1).

**Table 1:** Interviewees by clinician type

Clinician group	Number of interviews
GPs working in OOH service	4 (interviewed once) 3 (interviewed twice)
Emergency Practitioners: Advanced Nurse Practitioners (ANPs) (*n* = 3) and Paramedics (extended care practitioners (ECPs) (*n* = 3)	6
Ambulatory care physicians	2
Health Care Assistant (HCA)	1

Our findings are presented within three themes (described in Table 2), which relate to factors that clinicians considered when they were deciding whether or not to use POC tests, the ways in which POC test results were used to determine clinical management and gaining experience of POC tests.

**Table 2:** Themes and subthemes from thematic analysis

Theme	Sub-themes
1. Deciding whether to use POCT	-Is the patient or clinical situation “suitable” -Is the time/effort to do POC tests justified (what is the impact on resources?) -Will POC tests results reduce (or increase) my uncertainty and risk?
2. When undertaken, how can POC tests be used to make decisions about patient care?	- Determining the type (nature, location, timing) of onward care -Supporting professional communication
3. Gaining experience in using POCT	**-** becoming familiar with the equipment - gaining experience and confidence

### Theme 1. Deciding whether to use point of care tests

#### Is the patient or clinical situation “suitable”?

For many of the clinicians we spoke to, considering when and for whom POC testing was “appropriate” during OOH home visits was an important factor determining usage. Critical to this appraisal of “appropriateness” was whether they considered that POCT would add necessary or valuable information to their clinical assessment, or whether the results were likely to influence clinical care.

“*you know you're only doing it in cases where it's going to make a difference. So, for that reason, because I'm only doing it where it's going to either swing that decision one way or another, it actually really helps me. I'm not going to be using it where it's not going to make any difference” GP1, interview 2*

Within the group of clinicians we spoke to, we heard a spectrum of viewpoints about the value of the POCT available to them, ranging from thinking they had no significant role in out of hours care to considering them a valuable tool which completed a robust clinical assessment. Making complex assessments in patients’ homes and managing uncertainty, usually without prior knowledge of the patient or any background information, were central challenges of working in the out of hours home visiting service. For some clinicians, having access to more information to help them make care decisions for patients with complex multi-morbidities was a significant improvement. An example of the way in which this information could be used was in supporting safe prescribing in out of hours care.

*you know I always shy away from using nitrofurantoin because I don’t know people's renal [function] Whereas if you … .. knew the U&Es were OK, then it might be something you'd then use” GP1, interview 1*

While there were contrasting views on the place for POCT in clinical situations where there was uncertainty or an acute assessment was being made, there were some clinical scenarios within out of hours care where the POC tests were consistently considered to have a place. An example of this was when the hospital laboratory called the OOH team about abnormal blood potassium results. These results were usually from samples taken at GP surgeries earlier that day which were reported out of hours and then called through to urgent care. Without POCT, patients would typically have to travel to a hospital for assessment and repeat testing, however, POC tests allowed the potassium to be re-checked at home, either saving the patient a journey to hospital, or supporting the necessity of a hospital admission for an abnormal result.

#### What is the impact on resources?

Another important consideration for clinicians was how long the tests would take, in the context of the pressures of the workload in out of hours care. This included thinking about the impact of taking the time to do POC testing on the patients on their list yet to be assessed and the impact on the wider team. This constant awareness of the pressures of time and workload meant that the likelihood that the results would influence management was weighed up against how long it would take to get the blood, use the machine, and interpret the results. An example of this in practice was when a patient might have needed admission to hospital for blood tests, for example to check for an acute renal injury. In this context, POC testing could avoid the need for a lengthy phone call to arrange an admission if normal.

This awareness of the pressures of time made the practicalities around taking blood and using the machine important considerations for many clinicians, including the potential challenges of using the equipment in patients’ homes:

*“fiddliness and time – five to fifteen minutes roughly You know, … I don’t like to do it in the patient bed; I need a space “GP4*

*“it's potentially half an hour or something, and also … quite often … .with bad lights and cluttered surfaces” GP7*

One GP expressed a concern that doing POC tests might become an expected part of out of hours assessment, or that the capacity to use POC tests might be “abused” by in hours services, which could put a strain on out of hours or urgent care:

*“other GPs may say, "Oh call out of hours service. They ask them to come out to see you and they may do a blood test." So, when the people they know about it say, "Oh the GP who comes out can do blood tests," so they expect these things from them” GP2, interview 1*

#### Will POC tests results reduce or increase clinicians’ uncertainty and risk?

Clinicians’ assessments of whether the POC tests were likely to be useful were often under-pinned by their expectation that the results would increase or decrease the level of risk in their decision making. Out of hours home visiting was experienced as potentially high-risk clinical work. For some clinicians, POC tests increased their confidence or offered objective validation for their clinical assessment and decision-making:

*“you can only take so much from sort of visual and your assessment skills. Actually, it's quite good to just get a broad overview of what their electrolytes are doing you know … . it's quite good to just have those readings just to add sort of justification behind your decision making out in the community” EP6*

However, other clinicians wondered whether POC test results could potentially increase their clinical uncertainty or risk, for example because of incorrectly interpreting results, or not taking action on borderline results for patients who subsequently became more unwell. This included making clinical decisions about both blood tests that they had chosen to do themselves or when they were acting as advisers to other team members who had used POC tests.

*It's being comfortable with when you get your blood test results; what to do with them, isn't it, is the other thing. GP6*

*[A] s GPs we carry a lot of risk, and that I think is the bit of our job that GPs do that other specialists can't do, which is in dealing with that uncertainty. And you know the blood tests, I don’t know would necessarily help in that, and that’s the feeling I get from listening to the other clinicians. GP 5, interview 1*

The types of tests available in this project inevitably influenced clinicians’ perspectives on the necessity or value of using them. Clinicians reflected on what tests they might want to have had access to. Many of the clinicians identified markers of infection (such as CRP or white cell count in a full blood count) as desirable, although some expressed reservations. Again, these were largely focussed around uncertainties about the utility in clinical decision making and balanced against potential risks. This ambivalence was exemplified by contrasting views on CRP testing, where some clinicians felt the test added value as marker of severity of illness to guide admission decisions:

*“you know the other thing is the sort of CRP, you know that for acute infections and things. So, if someone's got a chest infection you want to think, 'OK, are they really poorly?' You know, you're trying to make that decision whether you can leave them at home.” GP 6*

Whereas others felt that a single CRP test without an understanding of trajectory added little value:

*everyone who's got some sort of illness will have a raised CRP. And the question is … you know if it was up at ten or twenty, that’s significant, whilst if you knew the trajectory was, you know fifty yesterday and it's ten today – great. But if it's ten now but in a few hours' time it's going to be fifty or a hundred, you can't know that” GP5, interview 2*

### Theme 2: when undertaken, how can POC tests be used to make decisions about patient care?

#### Determining onward care

When clinicians had chosen to use POCT, they reflected on the ways they had used the results to influence patient care. This included decisions about *where* onward care would happen, *what types* of onward care might be appropriate and *when* it was needed

*“there's certainly been one case that I can remember that, we had the blood results and … , they said, “ they're slightly abnormal but we think, you know it can wait," and I think then rather than send them in overnight … I think they arranged to go into AAU (acute ambulatory unit) the next day” EP4*

*“it gives you more sort of clarity as to, you know how unwell the patient is.. … ' You might think, 'Oh, they're fine,' and then you might do the bloods and actually, you know they’ve got a really high creatinine, and you think, 'Well actually, they're not fine,' and you know they do need to go in”. EP4*

#### Supporting professional communication

Both community and hospital interface practitioners told us that POC tests could provide valuable information to inform onward care journeys, even when a patient was being admitted to hospital. Examples included using a raised lactate to alert the receiving hospital team to suspected sepsis or facilitate initial management. The extra information from POC testing could facilitate communication both within the OOH team, and between the OOH and hospital teams, including helping clinicians feel confident when advocating for admission. The ability to have an informed focussed conversation was appreciated by both interface care and OOH clinicians:

*“It's critical because it allows you to kind of focus and we can say, "Look, we've done this, this, this and that’s alright, but there's still this one outstanding question." AAU1*

*“I think that any information that you can give them that will help them to prioritise or to tell you what they want; where they want the patient to go to … ..that’s got to help them with their bed management, but also for the patient's safety” EP5*

Whilst out on home visits, ANPs or ECPs could call an OOH GP for remote decision support. Whilst some GPs had concerns about sharing the decision making and risk for blood tests taken by a colleague in this situation, others found that it informed collaboration in developing a clinical plan:

*“it is really difficult for our doctors when you're speaking to them over the phone about a patient and, you know you're relaying information and they haven't got that patient in front of them; they can only go on what you're saying, whereas if you give them some actual, hard evidence .. … that is what the blood result is”. EP6*

#### Facilitating shared decision making

The extra information from POC tests supported clinicians in negotiating shared decisions with patients and their families, for example, when knowing the blood results identified more options as potentially safe or acceptable to the clinician and patient:

*“we were able to support what he wanted; that he didn’t need to go in acutely; we just needed to keep an eye on him and then we got him to agree that, 'Look, OK we agree with you, your bloods are OK-ish and your observations aren’t great but we'll agree that we'll come in and keep an eye on you over the next couple of days if you agree if things get worse you will go in. … and then ultimately it saved an admission” EP2*

### Theme 3: gaining experience of POC tests

#### Becoming familiar with the equipment

As clinicians became familiar with the equipment, it became easier to use. Systems to ensure that in-date cartridges were available and that barcode technologies were working were important facilitators for POC test use uptake. Having system level support, for example, guidance for using the machine, regularly delivered team training, and systems to ensure that all the equipment was routinely taken out on visits were identified as important supports for effective implementation.

*“once you’ve got the hang of it, I mean it doesn’t actually take that long, so you know really short, I mean ten minutes probably from start to finish” EP4*

#### Learning from clinical cases

Learning from colleagues’ experiences of using POC tests was identified as a useful training tool. Sharing learning about when it had been helpful or changed management at team meetings or in team newsletters was a suggested approach.

*“Definitely, a little case history of something, it would be useful wouldn’t it to know what situations they’ve used it in, and has it been useful, you know”.GP6*

During the initial phase of the service evaluation, some clinicians expressed concerns about the impact of testing on the pressures on the service, for example generating work and uncertainty in response to large numbers of indiscriminate tests done for uncertain indications. However, as the service evaluation progressed, these anxieties seemed to abate, largely because of the proportionate and careful approach that practitioners were taking to POC testing:

*“My fear was that it would be used like a blunderbuss and start getting people saying, "Oh this lady, have done her bloods and this has come up." And I'd be saying, "Well, why have you done them in the first place?" But that hasn’t been happening. So, yeah I think if it's used where it's going to make a difference, I think it can be very powerful … .I find it very useful and I think if it did go now there are cases where I'd be cursing that it wasn’t available. GP1, interview 2*

## Discussion

### Summary of findings

A key area of reflection for the clinicians was how, when and why they decided to use the POC tests that were made available to them during OOH home visits. These included considering which clinical scenarios were “suitable”, the balance of resources and time taken to do POC tests in the context of perceived likely clinical benefit from doing them, and potential implications for managing clinical risk. Once the decision to use POC tests had been made, clinicians told us how they had utilised the results, including planning onward care trajectories, and facilitating communication, both between professionals and with patients and their families. Gaining experience in using the equipment, and understanding where it had been clinically useful, supported further usage.

### Comparison with other literature

The value of POC test results to enhance inter-professional communication in primary care home visiting was a key theme in our study. The importance of team communication and trust to support the introduction and normalisation of POC testing into a medical base unit has been previously described [11].

The clinicians we interviewed expressed an interest in having access to POC tests to support diagnosis, treatment and communication in primary care OOH home visiting. However, this was tempered by concerns about the cost of POC tests, including the impact on the service of time taken to do the POC tests, what they add to the value of clinical judgement, challenges about result interpretation and managing patient expectations. This resonates with qualitative work with GPs about the potential introduction of CRP testing to guide antibiotic prescribing into primary care, which identified concerns about time implications, test accuracy, and potential risk [12]. A systematic review of qualitative studies exploring clinician attitudes towards point of care testing identified the central importance of considering the likely impact of POCT on diagnosis and decision making, with concerns identified about whether they undermine clinical skills, their costs and limited usefulness which clearly resonates with our findings [13].

### Implications for research and practice

In our study, clinicians chose to use tests only where they would add benefit, and initial concerns about indiscriminate testing were not realised. This is a useful insight for other urgent care services considering introducing this type of technology, as it suggests that practice is only likely to change to include POC tests in cases where this is needed, either for clinical confidence, or decision-making, or both.

We highlight a number of benefits of making POC testing available during OOH home visit consultations which were not easily measurable in a quantitative evaluation of their impact – including clinician confidence in decision making, in communicating with other parts of the healthcare system, and facilitation of shared decision making. This additional knowledge gained from our qualitative study is important for two reasons. Firstly, an implementation approach which emphasises these potential benefits could help to gain buy in from clinical teams in adopting this novel technology. Secondly, when weighing up the costs and benefits of introducing new technology, the possible benefits in terms of staff well-being are important for commissioners to consider. Developing appropriate educational resources to support implementation of testing was something highlighted by some of our clinicians as an enabling factor. Research to understand the key elements of this educational material is an important next step.

Studies of European OOH providers suggest that they are positive about potential benefits of POCTS [8]. Indeed, in Norway, rates of POC CRP use were shown to be higher in OOH than in in hours care [14]. However, to date there have been no comparable studies of a platform POCT in these settings, which is an evidence gap to be addressed. Which POCT will be of value to clinicians, and how and when they will be used are important emergent research questions.

### Strengths and imitations of this project

This is the first study to explore clinician experiences and beliefs surrounding the real-world introduction of POC tests into an OOH home visiting service, without supporting protocols, and with clinicians given free choice about when they utilised the equipment. Being able to speak to clinicians throughout the implementation journey allowed us to hear about how perspectives could evolve with experience and exposure. We interviewed a small group of clinicians who were interested in speaking to us about POC testing; and our sample included those both who had, and had not, chosen or been able to use the POC tests. Our sample was drawn from the limited pool of clinicians who worked in the two bases of a regional OOH service. However, we were able to include clinicians with different roles and a wide spectrum of usage including practitioners from the secondary-primary care interface which added perceptions of the potential role for of POC testing along the patient journey.

## Conclusion

In this qualitative service evaluation, clinicians identified a role for POCT in supporting them in decision making during OOH home visits but felt that they were not a panacea for all clinical decisions or dilemmas. Key uses were in assessing electrolyte and renal status, and in facilitating care planning and enhancing communication with both patients and professionals.

Initial fears that they would be excessively or indiscriminately used were not borne out. Clinicians were reflective about when they were necessary or helpful and were mindful of potential risks as well as benefits.

## Data Availability

The qualitative data is not available for open access. Please contact the corresponding author with any queries.

## Abbreviations

POC: Point-of care
OOH: Out of hours
EP: Emergency practitioner
ANP: Advanced Nurse Practitioner
ECP: Emergency Care Practitioners
AAU: Acute ambulatory unit
